# The Role of Low Complexity Regions in Protein Interaction Modes: An Illustration in Huntingtin

**DOI:** 10.3390/ijms22041727

**Published:** 2021-02-09

**Authors:** Kristina Kastano, Pablo Mier, Miguel A. Andrade-Navarro

**Affiliations:** Institute of Organismic and Molecular Evolution, Faculty of Biology, Johannes Gutenberg University of Mainz, 55128 Mainz, Germany; kkastano@uni-mainz.de (K.K.); munoz@uni-mainz.de (P.M.)

**Keywords:** low complexity regions, intrinsically disordered regions, compositionally biased regions, homorepeats, Huntingtin, protein interactions

## Abstract

Low complexity regions (LCRs) are very frequent in protein sequences, generally having a lower propensity to form structured domains and tending to be much less evolutionarily conserved than globular domains. Their higher abundance in eukaryotes and in species with more cellular types agrees with a growing number of reports on their function in protein interactions regulated by post-translational modifications. LCRs facilitate the increase of regulatory and network complexity required with the emergence of organisms with more complex tissue distribution and development. Although the low conservation and structural flexibility of LCRs complicate their study, evolutionary studies of proteins across species have been used to evaluate their significance and function. To investigate how to apply this evolutionary approach to the study of LCR function in protein–protein interactions, we performed a detailed analysis for Huntingtin (HTT), a large protein that is a hub for interaction with hundreds of proteins, has a variety of LCRs, and for which partial structural information (in complex with HAP40) is available. We hypothesize that proteins RASA1, SYN2, and KAT2B may compete with HAP40 for their attachment to the core of HTT using similar LCRs. Our results illustrate how evolution might favor the interplay of LCRs with domains, and the possibility of detecting multiple modes of LCR-mediated protein–protein interactions with a large hub such as HTT when enough protein interaction data is available.

## 1. Introduction

Huntingtin (HTT) is a large scaffolding protein (with 3142 amino acids in human) conserved in Bilateria, including Deuterostomia (e.g., humans) and Protostomia (e.g., *Caenorhabditis elegans*), but apparently not in Xenacoelomorpha (the most basal bilaterian clade) [1]. 

This protein is ubiquitously expressed in humans [2], however, a mutation by CAG trinucleotide expansion results in an expanded tract of consecutive glutamines (polyQ) in the *N*-terminal region, which causes a pathological effect in the brain resulting in Huntington’s disease, a neurodegenerative disease [3]. This suggests that the cell-type specific environment of the protein, including interacting proteins, must play a role in the disease.

As is the case with many structural proteins, HTT contains several low complexity regions (LCRs), which generally correspond to intrinsically disordered regions (IDRs) and could be involved in the modulation of protein interactions regulated by post-translational modifications (PTMs) [4]. Due to their flexibility, many such regions could not be solved in the recent resolution of human HTT’s structure in complex with HAP40 by cryo-electron microscopy [5].

The study of the function of LCRs is therefore necessary to understand protein functions, particularly protein interactions, but this is both experimentally and computationally complex due to their structural properties and low conservation [6]. Evolutionary approaches to compare LCR usage across selected sets of species have been used to categorize LCRs and learn about their significance and function in different contexts [7]. Here, we will illustrate how to examine LCRs in HTT and interactors, using a similar evolutionary approach to examine possible functions of LCRs in the interaction of HTT with partner proteins. HTT is a good protein for such a study because it contains a number of LCRs (some very well known, such as polyQ) and interacts with hundreds of partners [8]. 

Our hypothesis is that a large protein such as HTT may be able to have a few modes of LCR-modulated protein interaction shared by multiple partners; we might discern these modes by the presence of LCRs in HTT-interactors, which could even co-evolve with HTT LCRs. The recently solved structure of HTT in complex with HAP40 will also guide our study.

Our study should exemplify how to gain insight into LCR function in the context of interacting structural proteins, providing guidance for experimental work to study the function and regulation of HTT.

## 2. Results

### 2.1. LCRs in the 3D Structure of the Complex of HTT with HAP40

While human HTT (database record in UniProt: P42858) has already been known to be largely composed of alpha-helical tandem repeats since 1995 [9], it was not until 2018 that most of its structure was solved by electron microscopy (database record in the Protein Data Bank, PDB: 6EZ8, resolution 4.00 A; [5]). Incidentally, this was achieved in a complex with HAP40, signifying the flexibility of this protein and that its interactions may stabilize it thus making its study feasible. The interaction between HTT and HAP40 is conserved from human to (at least) fish [10]. 

**Figure 1:**
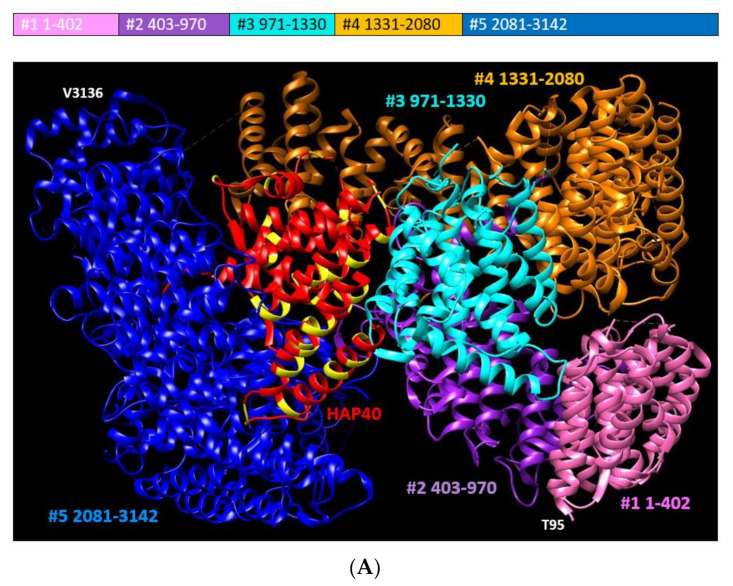

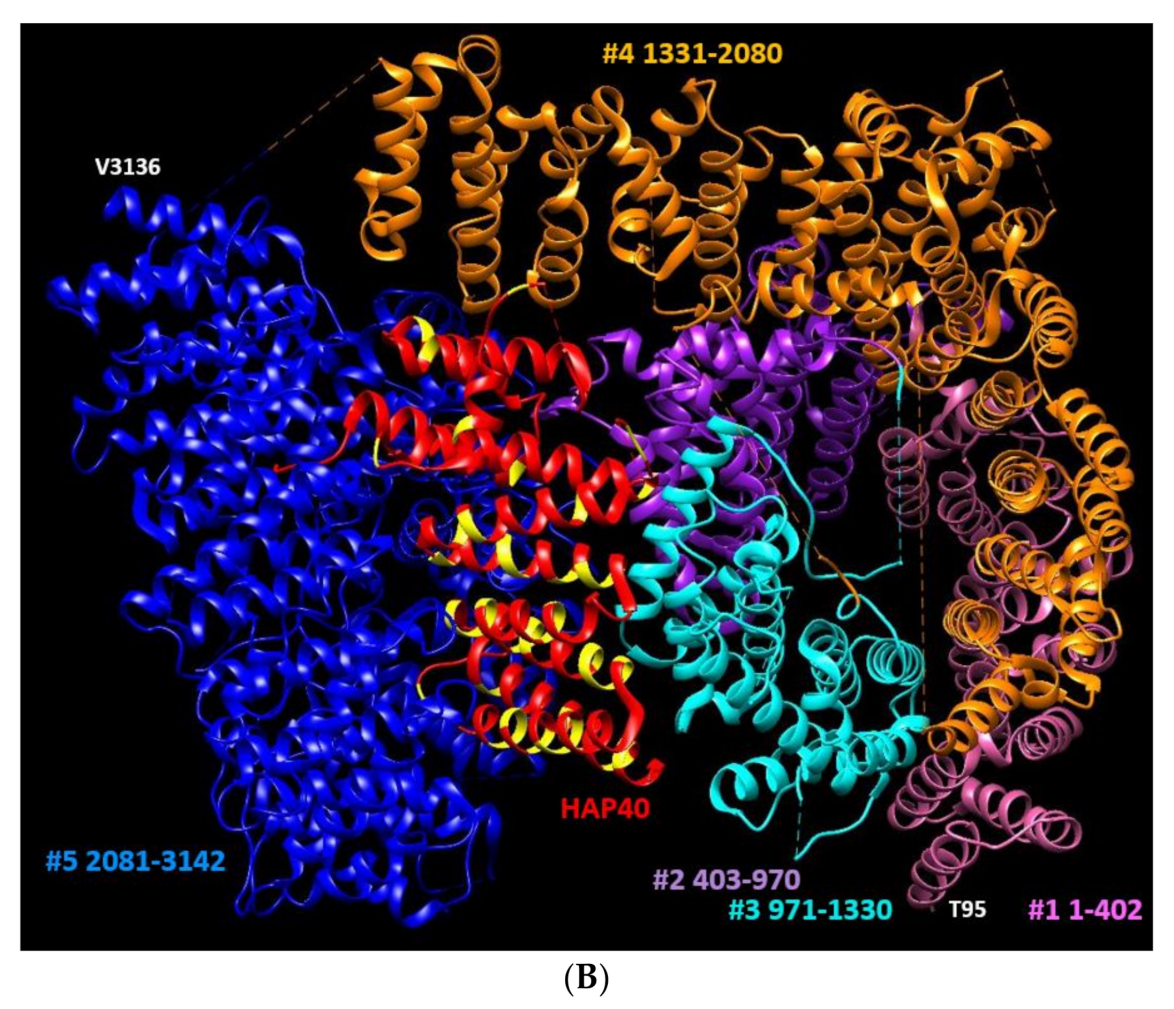
The structure of huntingtin (HTT) in complex with HAP40. From PDB:6X9O. Using disordered regions as borders, we defined the following regions of HTT: #1, 1–402 (pink); #2, 403–970 (violet); #3, 971–1330 (cyan); #4, 1331–2080 (orange); and #5, 2081–3142 (blue). HAP40 (red) is rich in alanines (yellow). The values of the HTT amino acid coordinates in the PDB record 6X9O are two units lower. The figure was generated using Chimera [13]. The views in (**A**,**B**) are rotated 90 degrees.

Currently, the only other structures of HTT (and of HAP40) available in the PDB are two more structures of the HTT:HAP40 complex solved by electron microscopy and without reviewed publications: PDB identifiers are 6RMH (9.60 A) investigating the effect of polyQ expansion [11] and 6X9O (2.60 A) [12] (Figure 1). We use the latter because of its high resolution. 

Regarding the 6X9O structure there are the following gaps in the structure of HTT that suggest disordered regions: 1–96, 407–665, 1165–1227, 1378–1422, and 2633–2662. Using these gaps, predictions of disorder (see later), and domain definitions from [5] and [1], we divided HTT in five domains (see caption of Figure 1 for details).

**Figure 2:**
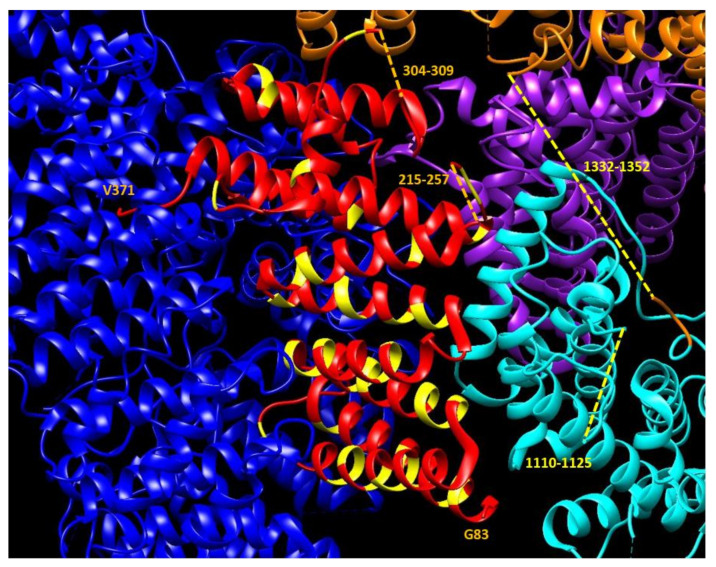
Detail of HAP40 bound to HTT. HAP40 interacts very tightly with the *C*-terminal domain of HTT (blue). The *C*-terminal V371 of HAP40 is inserted into the moiety of HEAT repeats. No disordered insertions are noted in this interface. Note the contrast with the interface of interaction between HAP40 and the HTT cyan domain. The side facing the viewer contains two unmodeled regions in HAP40 (orange dashed lines) and two in HTT (yellow dashed lines). These intrinsically disordered regions (IDRs) are likely to interact in 3D. All alanines in HAP40 have been colored in yellow to note that the *C*-terminal A-rich region belongs to the part of HAP40 facing away from the core of HTT.

HAP40 is tightly bound to the core of HTT, but there is a clear difference between the interface of HAP40 with the blue HTT domain (no insertions) and its interface with the cyan HTT domain (possibly containing interacting insertions). For HAP40, the gaps suggesting disordered regions are at positions: 1–82, 215–257, and 304–309. Two of these are situated close to disordered regions in the cyan HTT domain (Figure 2).

Taken together with the presence of an A-rich region in the *C*-terminal half of the HAP40 molecule (see alanines in HAP40 colored in yellow in Figure 1 and Figure 2), these observations suggest a mechanism of interaction between these two proteins that starts with the tightly fitting interaction of the *C*-terminal of HTT with the *C*-terminal half of HAP40, followed by a closing of HTT on the hydrophobic part of HAP40, with the cyan HTT domain grasping two disordered regions of HAP40 using its own disordered regions. This set of interacting flexible fragments in HAP40 and HTT can then be used to detect signaling events via PTMs controlling the opening and closing of HTT on HAP40.

Since structural information is lacking for many of the LCRs in HTT and HAP40, we will present approaches to study the function and conservation of LCRs in HTT and in HAP40 and other proteins that interact with HTT. We are interested to discover (i) commonalities in the LCRs of multiple proteins interacting with HTT that could indicate similar LCR-modulated modes of interacting with HTT; (ii) the co-presence of LCRs in HTT interactors that could indicate mechanisms of interaction requiring the coordination of multiple LCRs; and (iii) to find out co-evolution of LCRs in HTT and interactor partners that could hint at their direct physical interaction.

### 2.2. Prediction of LCRs in HTT-Interactors

We obtained a list of human proteins interacting with HTT from the HIPPIE database of human protein–protein interactions, scored according to the reliability of the experimental evidence for the interaction (HIPPIE v2.0; [14]). The current version of the database reports 402 HTT-interactors (including HTT itself as a self-interactor) with links to the manuscripts that report the experimental evidence of the interactions. 

The enrichment of functions in this set (see the Methods section for details) reports many terms related to protein interactions (e.g., the top one is “protein binding”, but see also “protein complex”, “identical protein binding”, “protein stabilization”, and “transcription factor binding”), which suggests that the function of HTT as a structural hub is also shared by its partners (terms with *p*-value < 1 × 10^−5^ in Table 1; see Supplementary File S1 for details). Other terms hint at the multiple subcellular locations and organelles that HTT might coordinate (e.g., cytosol, nucleoplasm, mitochondrion, membrane, extracellular exosome, autophagosome). 

Although HTT is widely studied in a neural context, it is noticeable that “myelin sheath” is the only neural-specific function, and protein degradation terms (e.g., ubiquitin protein ligase binding) and functions related to cell–cell adhesion (e.g., cadherin binding involved in cell–cell adhesion) are also present. This is consistent with the role of HTT in clathrin-mediated endocytosis, vesicle transport, cell signaling, morphogenesis, and transcriptional regulation, with a localization in nuclei, cell bodies, dendrites, and nerve terminals, and ubiquitous expression with the highest levels found in the nervous system, accordingly with functions in neuronal transport processes, post-synaptic signaling, and neuron protection from apoptosis [8].

Collecting predictions of LCRs in the set of proteins interacting with HTT and in their orthologs requires the making of two careful decisions: (i) the selection of a set of species as a reference set to find orthologs of HTT and interactors, noting that the orthologs might not interact in the corresponding species, and (ii) the selection of a set of LCRs and the protocols to predict them: here we decided on coiled coils (CCs), because they are involved in protein interactions, intrinsically disordered regions (IDRs), compositionally biased regions (CBRs), and homorepeats. 

CCs are a structural motif with a seven amino acid repeat resulting in alpha-helices that use hydrophobic residues to form homo-dimers or hetero-dimers with other coiled coils [15]. Although CCs adopt structure when they multimerize, they are disordered in monomeric state, which evidences their intrinsically disordered character [16]. IDRs are defined by their lack of fixed structure and can be predicted in sequence by analyzing the propensity of consecutive amino acids to result in interactions stabilizing protein structure [17]. CBRs are regions that have an amino acid composition that differs from average, and are usually characterized as regions rich in a particularly abundant amino acid [18]. Homorepeats (or polyX) are consecutive stretches of the same amino acid [19].

These LCRs overlap in their definitions [6]. For example, CCs often have reduced types of amino acids and have repetitive properties that make them similar to compositionally biased regions [20]. Both CBRs and homorepeats can be of 20 types, one for each amino acid, while CCs and IDRs are not specifically associated to any amino acid. Generally, homorepeats are shorter (10 to 40 amino acids) than CCs, IDRs, and CBRs, which can reach lengths above 100 amino acids.

The choice of species is very relevant because the taxonomic levels included define which variations in LCRs can be detected. In this exploratory analysis we decided to cover two fish and eight Tetrapoda species (including two rodents and two Sauria) to have the full range of emergence of the mammalian *N*-terminal polyQ, without going too far in the vertebrate lineage. The four pairs of taxonomically related species (fish, Sauria, rodents, and primates) allow for the detection of conserved features at independent taxonomic ranges, which are important to discriminate functional types of LCRs.

**Figure 3:**
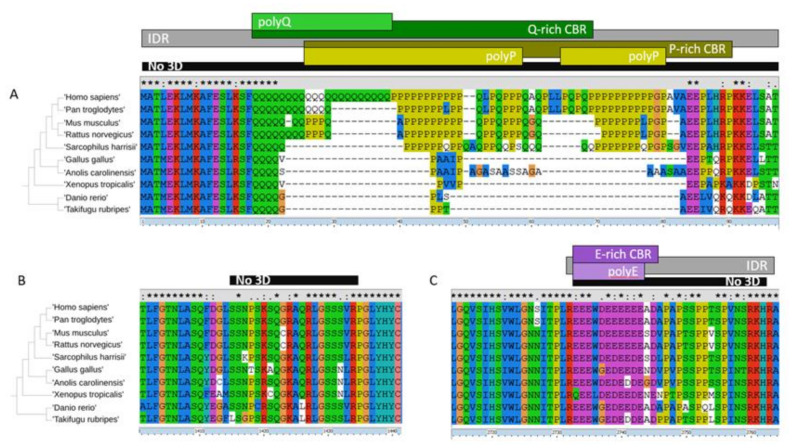
LCRs in HTT. Fragments of a multiple sequence alignment of HTT orthologs in 10 species. All four polyX detected in HTT are in the fragments shown. The multiple sequence alignment was represented using ClustalX [21]. (**A**) Human HTT (P42858) positions are 1–95 (not resolved in the 3D structure). (**B**) Positions 1318–1358, between the cyan and the orange domain (Figure 1 and Figure 2). (**C**) Positions 2613–2664, within the blue domain. Coordinates shown are from the alignment. No coiled coils (CCs) were detected for HTT orthologs. Sequence identifiers and LCR coordinates and alignments are available as Supplementary Files.

**Figure 4:**
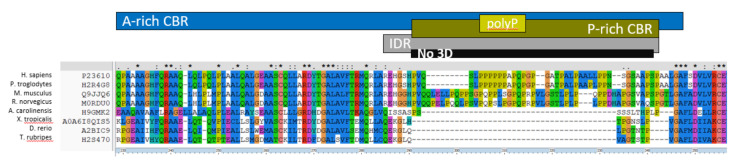
A-rich region and bordering polyP in HAP40. The polyP (alignment coordinates 305–314) overlaps a larger P-rich compositionally biased region (CBR) (291–342), which almost corresponds to a predicted IDR (285–342). These features are within the *N*-terminal of an A-rich CBR that extends well into the ordered part of HAP40 (111–347). The IDR prediction corresponds well to a fragment missing in 3D (indicated also in Figure 2).

**Figure 5:**
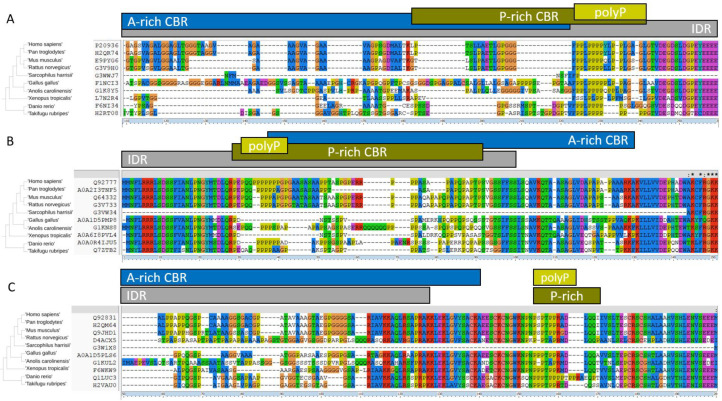
Conserved A-rich regions and bordering polyP in three HTT-interactors. (**A**) RASA1 (P20936). The polyP sequence (alignment coordinates 224–241) overlaps the end of a larger P-rich CBR (183–241), and occurs just after a much larger *N*-terminal A-rich region (3–222). All these LCRs are within a larger predicted IDR (1–263). (**B**) SYN2 (Q92777). The polyP sequence (31–42 in alignment) is within a larger P-rich CBR (29–91) and overlaps the start of an A-rich region (38–130). PolyP and P-rich regions are within a predicted IDR (1–100) but the A-rich region is partly outside. (**C**) KAT2B (Q92831). The polyP (154–164), above the threshold for *D. rerio*, overlaps a slightly longer P-rich region (154–169). They are near the end of a large *N*-terminal A-rich CBR (9–140), which overlaps with an IDR (8–127). Some of the sequences in the alignments may miss fragments and could have been replaced by better versions, but we have left them as given for simplicity (see Discussion).

The list of species used is visible in Figure 3, Figure 4 and Figure 5 and in the Methods section. The details about how LCRs are calculated are provided in the Methods section. Importantly, we describe the presence or absence of each LCR per protein across the 10 species. This requires the consolidation of LCRs from different sequences that overlap in the alignment. See details in the Methods section.

The identifiers of the orthologs used and other information attached to the groups are available in Supplementary File S2. The sequences of HTT and interactors used and the orthologs and alignments are provided as supplementary material (Supplementary Files S3 and S4, respectively), as well as the tables with LCR features predicted and their coordinates in the alignments: raw (Supplementary Files S5–S8) and consolidated (Supplementary File S9). Note that HTT interacts with itself and therefore its data is together with other HTT interactors.

The number of consolidated features detected is reported in Table 2. Note that it is possible to have multiple occurrences of the same LCR type (e.g., CCs, IDRs, or polyQs) in the same alignment, and that LCRs of different types can overlap; this happens often since, as mentioned before, their definitions overlap. 

We found an average of six LCRs per alignment: in total 236 polyX, 422 CCs, 658 IDRs, and 1035 CBRs. In general, CBRs of a given type were more frequent than the corresponding polyX, with the exception of L-rich CBRs and polyL (found in 4 and 11 alignments, respectively). Note that these LCRs are detected in alignments of orthologs, so they can be present in one or a few sequences and not necessarily in the human protein. We will study their conservation below.

To analyze if the LCR usage in HTT-interactors differs from other proteins, we computed the enrichment for the frequency of the LCRs found in the human proteins compared to their occurrence in the entire human proteome (Supplementary File S10). The enrichment in CCs is very significant (89 of the 402 interactors had them, compared to 2083 of 20609 proteins in the human proteome, *p*-value = 3.48 × 10^−12^), and that of IDRs modest (208 of HTT-interactors had them, compared to 9084 in the human proteome, *p*-value = 0.00233). 

HTT has a polyQ, an LCR that has been proposed to function in the modulation of interactions between CCs [22], and it is known that proteins that interact with polyQ proteins are enriched in CCs [23]. Regarding LCRs by type, E- and K-rich CBRs are significantly enriched (*p*-values = 2.72 × 10^−12^ and 7.65 × 10^−5^) with moderate enrichments of Q-, S-, and T-rich CBRs (*p*-value < 0.01). The only significantly enriched polyX we found was polyQ (10 out of 177, *p*-value = 0.00224), also consistent with the typical enrichment of polyQ proteins among proteins that interact with polyQ proteins [23]. 

Next, we illustrate the predictions obtained for HTT and HAP40, to understand how different LCRs overlap, how they correspond to the current 3D information that we have available, and how they may play a role in the protein interactions of HTT.

### 2.3. LCRs in HTT and HAP40: Categories, Overlap, and Conservation

Regarding the HTT alignment (with human P42858 as the leading sequence; group number #4; alignment available in Supplementary File S4), we detected 15 LCRs: zero CCs, four IDRs, seven CBRs, and four polyX (Table 3; also as entries for P42858 in Supplementary File S2). Some of these LCRs reflect known functional disordered regions in HTT and overlap each other. Most of them are absent from the 3D structure due to their flexibility. For example, the large fragment missing between HEAT repeats 6 and 7 (from positions 400 to 674 in human HTT) is quite accurately detected as a completely conserved (present in all ten orthologs) IDR and also as an S-rich CBR (Table 3; note that the coordinates there are from the alignment of the orthologs and will differ from the protein coordinates). We show three fragments of the HTT alignment #4 in Figure 3 to illustrate other disordered regions.

In Figure 3A we see the *N*-terminal region of HTT, which contains in the human sequence the polyQ whose expansion produces a genetic disease that manifests by protein aggregates. The polyQ followed by polyP has a function in the modulation of protein interactions mediated by coiled-coil interactions [23]. The polyQ prolongs the preceding alpha-helix in an *N*-terminal gradient where closest residues adopt the helical conformation more often [24]. The polyP overlaps a proline-rich domain (PRD) that adopts a stiff structure thought to reduce the aggregation propensity of the preceding polyQ [25]. This PRD was noted in HTT [26]. Its structure has been studied in the context of disease caused by the expanded polyQ but it has a function and participates in interactions with a number of proteins [8]. The polyQ has been detected also as a longer Q-rich CBR and both overlap a polyP and a P-rich CBR (Figure 3A). These features (including the IDR encompassing them all) are mostly detected in five of the orthologs (mammalian species). 

In Figure 3B we focus on a small region that is absent from the 3D coordinates, and thus likely disordered, but was not detected as an IDR. This region is too small for the window used and indicates the limits of the detection method. Had there been an insertion present in any of the orthologs making them longer, it would had been identified. On the contrary, in this set of species the length of this region is completely conserved: there are neither insertions nor deletions in the alignment. This region is between the cyan and the orange domain (Figure 1) and could be in contact with unresolved disordered regions from HAP40 (Figure 2).

The third fragment we showcase is another strongly conserved LCR with an E-bias (Figure 3C) corresponding to HTT human coordinates 2614–2664 within the blue domain (Figure 1) and facing away from the pocket holding HAP40. As far as we know, this has not been noticed before and it is not known to have a function, but its conservation suggests that it must be functional. It is predicted to be part of an *N*-terminally extended IDR and this is precisely matched by the absence of 3D information (Figure 3C). The slight lower frequency of glutamic residues in both fish species makes the detection of the region fall to just below the thresholds used for detection (see patterns of conservation in Table 3 for features in alignment coordinates around 2732–2763). However, manual inspection of the alignment indicates that the acidic character of the region is conserved with some aspartic residues in place of the glutamic residues observed in Tetrapoda species (Figure 3C).

We discuss now the LCRs detected in HAP40 to contrast them with the structural information that we have from HAP40 in complex with HTT. HAP40 (UniProt: P23610) has a HIPPIE score of 0.73 (>0.72; high confidence HIPPIE score category) for its interaction with HTT. We have an alignment for orthologs in eight of the ten species (missing *S. harrisii* and *G. gallus*; alignment #6 in Supplementary File S4). We detected five LCRs in this alignment (Table 3). The most significant ones are encompassed in the fragment shown in Figure 4: one IDR, a more extended conserved A-rich region, an overlapping P-rich region, and a shorter overlapping polyP. The A-rich region corresponds in the human sequence to VAEAG-AALGA (coordinates 43–261) including a polyP “PPPPPPAPQP” (coordinates 223–232). The P-rich region is missing from the 3D structure (Figure 2) and it is likely flexible. It is far away from the *N*-terminal of HTT and possibly not competing with its polyP.

In the complex between HAP40 and HTT, this disordered P-rich region in HAP40 is close to several disordered regions in HTT (Figure 2), including the one we depict in Figure 3B. This HTT region is serine-rich. Serine phosphorylation is a mechanism by which protein interactions are regulated and this occurs particularly in IDRs, which are appropriate for this regulatory function and modification since they are often exposed [4]. Regulation by serine phosphorylation is already known for HTT. For example, CDK5 phosphorylates serines at positions 1179 and 1199 in response to DNA damage [27]. These serine residues happen to be in a region missing from the 3D structure within the cyan domain (human HTT coordinates 1162–1226) and are predicted to be a conserved IDR overlapping a conserved S-rich CBR (Table 3; alignment coordinates 1245–1305 and 1033–1312, respectively).

Apparently, this polyP in HAP40 is dispensable for interaction with HTT [5], reflecting the fact that it is not in the interface between these two proteins and seems to be flexible even when they form a complex. However, it might have a regulatory role. The more disordered regions are used to close and decorate an interaction, the more complex the combination required to unlock the interaction could be. The interplay between disordered regions proximal in 3D from two interacting partners is likely to provide an explosion of combinatorial possibilities for regulation.

### 2.4. Different Modes of Interaction with HTT

Since HTT is a large protein, there can be different modes of interaction with it, and HAP40 could be an example of one of them. If LCRs are used in these interactions, we could detect other proteins binding like HAP40 if they have a similar profile of LCRs.

Differently from HAP40, there are many interactors that interact with the *N*-terminal of HTT. This is relevant to the pathogenicity of polyQ-expanded HTT because these interactors increase the size of pathogenic aggregates. This is an effect that has been observed for multiple proteins and can be explained by the spoiling effect that the expanded polyQ has in the modulated coiled-coil interaction [28]. Proteins that bind HTT elsewhere may not affect the formation of aggregates and may even reduce the aggregates because they can sequester the toxic protein from the aggregates instead of contributing to them. This effect has been demonstrated in a different polyQ expanded protein: a toxic construct of Ataxin1 [29]. 

The PRD is a relevant feature in the *N*-terminal of HTT and a few proteins are known to interact with it [8]. Some of them do so with WW domains [30] and some with SH3 domains [31]. Interestingly, HTT-interactors are very significantly enriched in WW and SH3 domain-containing proteins as both of them are domains that interact with P-rich regions [32]: 11 interactors vs. 53 in background (*p*-value = 4 × 10^−9^) and 23 interactors vs. 225 in background (*p*-value = 7.34 × 10^−11^), respectively. No HTT interactor has both domains (in the entire human proteome only four proteins have both SH3 and WW domains) but each of these domains tend to occur more than once, often in tandem. This multiplicity of domains binding proline-rich regions, together with the presence of competing proline-rich regions in the sequences themselves (see e.g., the P-rich region modulating the interaction of WW domain-containing protein HYPB/SETD2 with the PRD in HTT [33]), suggest that interactions between multiple LCRs and the domains recognizing them can result in very rich and complex regulatory patterns. Interestingly, the HTT-interactors containing WW/SH3 domains have an average HIPPIE score of 0.72, which is a high confidence level, and is above the 0.66 average value of all HTT-interactors.

The PRD has a 1111100000 conservation profile (Figure 3A), which suggests that HTT gained interactions with WW and SH3 domain-containing proteins within the mammalian lineage. Since this domain is not conserved in evolution, this sets the question of whether the WW proteins that interact with human HTT do so in species that lack the PRD.

### 2.5. LCRs in HTT Interactors: Conservation, Abundance, and Co-Occurrence

To look for groups of HTT interactors using similar LCRs we need to evaluate (i) their levels of conservation, (ii) which types of LCRs are (unusually) frequent among HTT interactors, and ultimately (iii) try to find if there are some that occur together and could reveal LCR-regulated modes of interaction. The data we present in the next paragraphs reports the frequency, composition, and conservation patterns of LCRs in HTT interactors, and we will use these data to guide our search for LCRs that could work together for interactions with HTT among those unusually frequent and similarly conserved in HTT interactors.

The general conservation patterns by feature are described in Supplementary Figure S1. For CCs, IDRs, and CBRs, the most frequent pattern is full conservation, particularly for IDRs, which appear to be the more stable type of LCR, suggesting that, in the taxonomic sampling of 10 species chosen for this study, polyX is the evolutionarily more dynamic LCR. A total of six polyX conservation patterns are found 10 or more times showing specificity for the most distant species: each of the fish, for both of them, for *A. carolinensis*, *X. tropicalis*, or for *S. harrisii*.

Interestingly, the set of HTT interactors containing at least one fully conserved LCR has a higher average HIPPIE score than the full set of 402 interactors (values of 0.71, 0.69, 0.69, and 0.72 for CCs, IDRs, CBRs, and polyX, respectively, versus 0.67 for the full set), suggesting that IDR conservation is associated with a reliably measured interaction.

It is known that there are differences in the conservation patterns of CBRs [7] and polyX [34] depending on the type of amino acid. We show the main conservation patterns by amino acid type in HTT interactors in Supplementary Figure S2.

Regarding CBRs, two of the most frequent (Table 2) are E- and S-rich CBRs, which show a clear bias towards full conservation (Supplementary Figure S2A). Other frequent CBRs are G-, A-, K-, Q-, and P-rich, with full conservation not being so separated from the rest of the patterns. These results agree with the general stability previously observed for E-rich regions and the more dynamic behavior observed for Q-rich regions [7]. Most of the CBR conservation patterns indicate conservation in one or very few species, indicating fast selection. Patterns where the LCR is lost in one species and conserved in the rest are rare, suggesting that once a CBR is gained it is difficult to remove it. These results indicate that CBRs are selected and functional. 

Regarding polyX, which were identified in fewer occasions than CBRs, the most frequent are polyE, polyP, and polyS (the same amino acids associated to top CBRs; Table 2). But differently to CBRs, the most frequent conservation patterns correspond to conservation in one species, rarely in more (Supplementary Figure S2B).

We computed the enrichment of LCRs by contrasting the number of proteins containing LCRs of each type found among the 402 human HTT-interactors with the background of 20,609 human proteins (see the Methods section for details). We note that the size of HTT-interactors is not significantly different from the size of other human proteins (614 to 551, respectively).

We found a significant enrichment in IDRs (208 have IDRs, *p*-value = 0.002), which tend to be, but are not necessarily, regions of low complexity [6]. Coiled coil regions tend to have a reduced number of amino acid types and are low in complexity but adopt alpha-helical conformation and are often involved in interactions with other coiled coils, thus they tend to be a motif for protein interaction [35] and this can be detected by their co-evolution in interacting proteins [36]. The enrichment in coiled coil-containing proteins is extremely significant among HTT-interactors (89 contain them, *p*-value 3.48 × 10^−12^). Considering that HTT has a mode of interaction via polyQ following the *N*-terminal helix, this is not too surprising, and we expect that many of these proteins interact with the *N*-terminal of HTT.

Regarding amino acid-rich regions, there is a very strong enrichment on E-rich regions (with 110 interactors containing them, *p*-value 2.72 × 10^−12^) and K-rich regions (53 interactors, *p*-value 7.65 × 10^−5^). E-rich regions were also often found to be conserved in all 10 species considered (Supplementary Figure S2A). Q-rich regions were also observed as enriched (40 interactors, *p*-value = 3.34 × 10^−4^) but were more variable among species (Supplementary Figure S2A).

Given the lower number of polyX identified, it was more unlikely to find significantly enriched polyX in HTT-interactors. The only one found was polyQ (10 interactors, *p*-value = 0.0022), which is also consistent with CC interactions with HTT and its polyQ. This association is found when examining the entire proteome (CC and polyQ tend to coexist [23]) but we could not find this association in HTT interactors given the low numbers (only 1 of the 10 HTT-interactors with polyQ was predicted to contain CC).

Next, we calculated the co-occurrence of features (IDRs, CCs, the 20 CBRs, and the 20 polyX) in HTT interactors and orthologs used in 10 species. Co-occurrence was calculated across all proteins by generating a vector of absence and presence in these proteins for each LCR and then computing the Jaccard score between all pairs of vectors (see Methods section for details). A score of zero means no co-occurrence between the pair of LCRs in any protein and a value of 1 represents perfect co-occurrence in all proteins. Obviously, LCRs that occur more often have a better chance of produce higher values than those that occur very rarely. However, for a given LCR the matrix of values allows us to find the ones it co-occurs more often with (Supplementary Figure S3).

Co-occurrence can be due to LCRs being detected for the same region (overlapping features). This explains, for example, the high score obtained for the co-occurrence of CCs with IDRs: CCs are often identified as IDRs reflecting their disordered nature when they are in monomeric state [37]. Beyond this high co-occurrence between IDRs and CCs, corresponding to a tendency to identify CCs as being part of IDRs, other high values of co-occurrence with IDRs probably due to overlap are observed for E- and S-rich CBRs, which are the two CBRs observed more frequently among HTT-interactors (Supplementary Figure S2A).

Co-occurrence due to overlap can also be expected between each polyX and the corresponding X-rich CBR. This is observed for the most frequent ones, such as polyG and G-rich CBRs (value of 0.25; Supplementary Figure S3). Regarding co-occurrence of different polyX, unlikely to result from overlap, the highest value corresponds to the known association of polyP with polyQ (0.10).

The scores of co-occurrence between different X-rich regions suggest other interesting pairings not due to overlap. The strongest signal is from E-rich and K-rich regions (0.24). Considering consolidated regions (Table 2), E-rich regions are the most frequent CBRs, but K-rich ones are less frequent than S-, P-, and Q-rich CBRs. Differently, P- and Q-rich CBRs seem to co-occur with S-rich regions (scores of 0.22 and 0.20, respectively).

If we compare the values of co-occurrence between IDRs (1st row) and CCs (2nd row), we can see that all values decrease (e.g., A-rich region co-occurrence with IDRs scores 0.08 and with CCs 0.05). The prominent exception are the Q-rich regions, which have stronger co-occurrence with CCs. This agrees with the role of polyQ in modulating CC interactions [23] that seems to extend in this context also to Q-rich regions. 

Since CC could be characteristic of the interaction mode with HTT *N*-terminal helix and polyQ of HTT, we can consider the most depleted CBRs in their co-occurrence with CCs as participating in a different mode of interaction. In fact, according to Supplementary Figure S3, A-rich CBRs have a high ratio of co-occurrence with IDRs versus CC (0.08 to 0.05), thus making them a good LCR candidate. Since polyP has one of the top co-occurrence scores of polyX with A-rich CBRs (0.06), it seems appropriate to search for HTT-interactors with these two features to propose proteins that could bind like HAP40. HAP40 has a conserved A-rich CBR followed by a polyP conserved in human and *P. troglodytes* that aligns to an apparently disordered region in the other species (Figure 4). We could then mine the data to identify HTT interactors with these LCRs as HAP40-like HTT interactors. 

Of the 402 HTT-interactor alignments, we identified 76 A-rich CBRs in 66 alignments. Since this could be an important feature for conserved interaction, we considered next A-rich regions conserved in at least four species, which reduces the list to 15 A-rich regions in 15 alignments. To ensure that they were diffuse as in HAP40 (as opposed to due to a polyA, that is, to a stretch of consecutive alanines) we removed those overlapping with any polyA (no matter how conserved it was); this reduced the set to 9 alignments. Interestingly, all 9 contained at least one P-rich CBR. To increase similarity to HAP40, we next required them to have a polyP (no matter how conserved), so that there were not just diffuse P-rich regions. Four of them had a polyP, that is, HAP40 and three other proteins. Interestingly, in all three cases the polyP was bordering the A-rich region, twice at the *C*-terminal, as in HAP40, and once at the *N*-terminal (Figure 5).

In human RASA1 (P20936), the A-rich CBR and its *N*-terminal polyP are in the *N*-terminal 150 amino acids of the protein, before the most *N*-terminal predicted domain, an SH2 domain (protein coordinates 181–272). In human SYN2 (Q92777), the A-rich CBR and its *C*-terminal polyP occupy positions identified as a linker (31–113) *N*-terminal from domains binding actin and synaptic-vesicles. In human KAT2B (Q92831) the A-rich CBR and the polyP are in the *N*-terminal 130 amino acids of the protein, well outside the *N*-acetyltransferase (503–651) and Bromo domains (740–810) known in this sequence. Our results suggest that these three proteins might use a similar mode of interaction with HTT as HAP40, consisting of a region rich in alanines (therefore hydrophobic yet relatively flexible, since alanine is an amino acid with a small side chain), flanked by a polyP that could be modulating their interaction with HTT.

### 2.6. IDR Coevolution of HTT and HTT-Interactors

In this last section of the results, we explore an alternative use of the correlation study between groups of orthologs focused on IDRs. Now, because we are going to use IDR predictions, we ignore the amino acid type, but become more precise by comparing the position of the IDRs in individual aligned sequences, that is, we will not be using the consolidated data. 

The main hypothesis remains: because many IDRs are involved in protein interactions, we hypothesize that the evolution of IDRs could be correlated (i) between proteins that interact using a similar mode of interaction, and additionally (ii) with the protein (or protein fragment in this case, see below) they interact with. For example, if a number of proteins interact with a region of HTT where some IDRs are inserted in evolution that require the parallel insertion (or deletion) of an interacting IDR in these proteins, then this should generate parallel variations in the IDRs of the corresponding species as well as parallel variations of IDRs in HTT. 

We previously applied this hypothesis to study the correlation between orthologs conserved across five metazoan species [7]. Here we apply this approach in a more focused way to find IDR correlations between HTT and the eleven WW-domain containing HTT interactors, to try to get information on the mode of interaction of these proteins with HTT.

Since HTT is a large protein, we considered the five HTT regions defined above separated by large IDRs (Figure 1). We wanted to find out if the HTT fragments behaved differently, and if we could discriminate between the putative WW-domain containing HTT-interactors and find where they bind to. Correlations in IDR variation between the HTT fragments and the interactors should point to the position of the interface of interaction in HTT.

**Figure 6:**
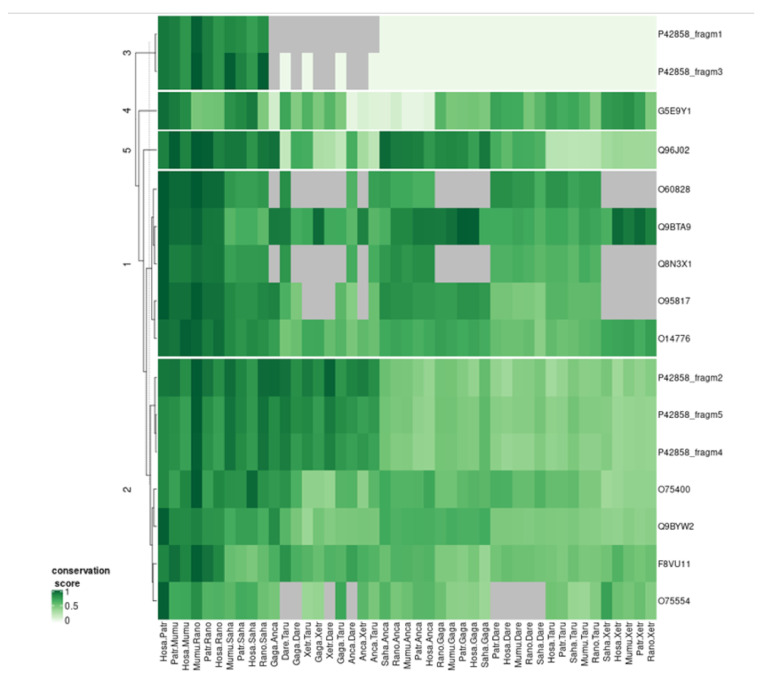
IDR co-evolution of WW proteins and HTT fragments. Each row represents the scores of overlap of the IDRs for each possible pair of orthologs for a given HTT interactor (UniProt ID) with WW domains, or for five fragments of HTT (P42858_fragm1/5). The data was hierarchically clustered. Five clusters are indicated. If one of the orthologs does not exist, a comparison is not possible and the corresponding cell is colored grey. If both sequences to be compared have no predicted IDRs, we consider the comparison not informative and the corresponding cell is also colored grey. Grey cells do not contribute to the clustering. See the Methods section for details. The heatmap was produced with the R package complex heatmap [38]. The values represented in the heatmap are available as Supplementary File S12.

HTT fragments and orthologs were clustered according to the scores of overlap between all possible pairs of species (see Methods section for details, Figure 6). HTT fragments 2, 4, and 5 clustered with four of the interactors. Three of these are among the top four of the eleven WW-domain containing HTT-interactors according to their scores in the HIPPIE database, indicating that this cluster detected interactors with the best experimental information reporting their interaction with HTT (average score of 0.83 versus and average score for the other seven interactors of 0.66). The one with the lowest score (0.63) was that of WBP4 (O75554), for which there is no specific publication describing experimentally its interaction with HTT.

Regarding the HTT fragments 1 and 3, they did not display much variability and lacked IDRs for some species, so they clustered together. Fragments 2, 4, and 5 had very similar profiles of variation and clustered together. This result suggests coordinated IDR evolution between the large domains of HTT but does not help to define a position for the interaction of the WW-domain containing interactors. It does suggest that WW domain interactors must interact with other HTT regions beyond their expected interaction with the PRD of the *N*-terminal of HTT. One could expect this to happen in the opposite side of the pocket admitting HAP40, which would allow these proteins to contact fragments 2, 4, and 5 of HTT (violet, orange, and blue in Figure 1). This could also mean that the interactions of these proteins with HTT are more stable (thus easier to detect) than those of the average interactor. In fact, three of these interactors were detected in an early yeast two-hybrid (Y2H) study [30] with ten other non-WW interactors, and received further support in the meantime from other studies.

## 3. Discussion

Evolutionary studies can be of help to elucidate general principles that drive the evolution of cellular function in terms of networks of interacting proteins. This approach was previously followed to study HTT interactors like HAP40 [10]. Here we expanded this approach to all HTT interactors and focused on LCRs since they evolve very fast and are related to protein interaction [6].

### 3.1. Studying Multiple Modes of Interaction with HTT

HTT is a very good example to study how LCRs can modulate multiple modes of interaction with a given protein because HTT: (i) is a large and flexible protein with many possible interfaces of interaction, (ii) contains several conserved LCRs of various types, and (iii) has many known interactors [8], which also have LCRs. In addition, many studies have focused on LCRs in HTT and how they function in the interactions of HTT with other proteins because of their possible involvement in disease and to search for therapeutic applications.

In the *N*-terminal of HTT we find the most known complex of LCRs with a polyQ, which emerged in Chordata, followed by a proline-rich domain (PRD), which emerged in Mammalia. A very conserved E-rich region located in a different part of HTT could be controlling a second mode of HTT interaction: this is a more ancient LCR. While fish have it, *Branchiostoma floridae* (Florida lancelet, amphioxus) does not have it (although it conserves the surrounding sequence; data not shown) indicating emergence within Craniata. Interestingly, the only structures of HTT currently available are in interaction with HAP40, which seems to contact a different part of HTT and a different set of LCRs, thus illustrating yet another mode of interaction with HTT.

### 3.2. LCRs and Domains that Bind Them: Partners in Evolution?

The evolution of the PRD in HTT poses an interesting question. P-rich regions that emerge as *C*-terminal to polyQ are known to occur in multiple proteins. This is a mechanism that avoids polyQ aggregation but at the same time provides a surface for proteins avidly seeking P-rich regions because they contain domains that bind them. These dependencies suggest that the emergence of domains that are capable of binding to particular types of LCRs that evolve with ease creates opportunities for evolution. The emergence of a new P-rich region in HTT offered a possibility for hundreds of proteins already having domains able to bind P-rich regions. Apparently, many of them adapted to this evolutionary modification of HTT and jumped on the bandwagon of HTT interactors, probably slightly adapting their existing SH3/WW domains and other interaction motifs to accommodate new interactions with HTT. In summary, this effect is possible both due to mechanisms that favor the emergence of new disordered regions, and to the emergence of domains able to recognize generally compositionally biased motifs.

It is clear that not all WW/SH3 domain-containing proteins bind all P-rich regions, but the increased emergence of P-rich regions in some lineages and the existence of such specialized domains facilitates quick adaptations to generate new interactions. These interactions can be tuned for specificity by using other parts of the protein or adapting the P-rich binding domain itself. 

The fact that this strategy is very favorable is made evident by the fact that, in an example of convergent evolution, not one but two domains emerged in evolution to fulfill a similar P-rich recognizing function. We hypothesize that there could be other domains or motifs loosely able to interact with particular LCR types. The investigation of LCRs involved in interaction interfaces should help to find them. For example, one of those domains could be the S-rich flexible region in HTT that we described to be close to a HAP40 P-rich region in the structure of the HAP40:HTT complex.

### 3.3. Different Modes of Interaction with Different Regulatory Relevance?

HTT is a hub for a multiplicity of functions, as indicated by the multiple functions and cellular locations of its protein partners. Regardless, it is possible that different modes of interaction with HTT might have a different role in the regulation of the function of HTT. 

The tight binding of a protein in a central position (like HAP40) could have a function in directing HTT to the selection of a few roles in different environments, while other binding sites more exposed (such as the site of the PRD, or the E-rich region) could have a modulatory role with more temporal, weaker interactions. This would also have a correspondence in their evolutionary emergence, with more relevant interaction modes being more ancestral and more lateral roles being of a more recent evolution.

A Y2H analysis of the interaction between HTT fragments suggested that there is a strong interaction between HTT fragments 507–1230 (violet and cyan domains in Figure 1) and 2721–3144 (blue in Figure 1) [1]. This interaction reflects a closed HTT structure without a pocket for HAP40 or other proteins. This would explain the need of a hydrophobic and flexible domain for a protein to insert itself in the HTT’s core, by competing with hydrophobic interactions. It is easy to picture that the extraction and insertion of proteins into this position could require much more energy than forming other interactions in the periphery of HTT, which could be more transient. It would be very interesting to test this hypothesis by solving the structures of the other three proteins in complex with HTT. If they bind similarly to HAP40, they could be expected to have a similar stabilizing effect on HTT.

What could be the functional relevance of HAP40 (F8A1), RASA1, SYN2, and KAT2B competing for their attachment to the core of HTT? The answer to this question is intimately related to their different functions. The interaction of HAP40 is well characterized structurally as this is part of the (only) known HTT structure. It mediates the recruitment of HTT to early endosomes [39]. The interaction between RASA1/RasGAP and HTT is reported in two papers, one of them specifically devoted to testing the interaction of HTT with SH3 domain-containing proteins related to tyrosine kinase receptor-mediated signaling; it was found that the SH3 domain of RasGAP is enough for the interaction [31]. For SYN2 (Synapsin-2) there is no specific study on its interaction with HTT, but it shares a neural function. Synapsins store synaptic vesicles attaching them to the cytoskeleton until they need to be used [40]. For KAT2B/PCAF, a specific study found its interaction with the *N*-terminal (HTTex1p) domain of HTT [41]. KAT2B has histone acetyltransferase functionality, but it is a large protein that could be multifunctional. 

The competition of proteins using the same mode of interaction with HTT could explain general mechanisms to coordinate separate pathways by making them to converge on a shared hub. The involvement of LCRs would be necessary to provide variable combinations of flexible linkers able to hold post-translational modifications, requiring a series of precise modifications for the binding and release of particular partners. It is difficult to imagine how to obtain this regulatory richness without the participation of LCRs.

### 3.4. Limitations of Our Study

Many of the sequences included missed fragments or had obvious mistranslated fragments, likely due to incomplete or wrong genome sequence assembly and gene prediction. This situation can be solved with manual work and we did this for the alignment of HTT orthologs given their importance for the present study. It is expected that this problem will be solved with time, since it is possible to find better sequences in the databases or compile them by joining fragments of predicted proteins using multiple sequence alignments as templates. We have tried to minimize biases given by this issue by paying attention to the features conserved in more than one species.

In the identification of LCRs, we have used strict thresholds to reduce false positive detection. For example, the three polyP illustrated in Figure 5 are not above the 8/10 threshold in all the species considered in the alignments, but the presence of variable but conserved stretches of consecutive prolines in all species can be visually identified. The type of analyses carried out here, which extracted information from hundreds of alignments, require automated procedures. Our results suggest that the thresholds used are accurate enough to provide significant insights into low complexity features enriched in HTT interactors. 

### 3.5. Conclusions

Many studies have focused on LCRs in HTT and interacting proteins because of their possible involvement in disease, or for therapeutic applications [8]. As we have noted before, this might have biased such studies towards the *N*-terminal region of the protein and away from functions that are not related to the neural context of the disease. We hope that our study will have a beneficial effect by raising awareness about the need to study interactions with other regions of HTT, as well as by paving the way for studies of LCRs in other large proteins with many known interactors. We expect that LCRs will be common in many protein interaction surfaces and their study will inform us of how LCRs interact with each other and how their interactions are regulated by PTMs. 

In addition, as we have seen with HAP40, it might be easier to study the structure of HTT (and of other large and flexible proteins) when they are stabilized by their interaction with a partner protein. In this respect, it seems very important to develop methods that propose examples of proteins and target regions of large proteins interacting with them, in order to facilitate the obtaining of high-resolution structures, and the evolutionary study of interacting LCRs seems a good approach to guide this process. Similar to a puzzle that becomes easier to solve as more pieces are assembled, we expect that as we discover more interactors and solve their structures in complexes by identifying the positions and types of LCRs involved in the interaction, it will become increasingly simpler to discover more modes of interaction modulated by LCRs and to discover additional interaction interfaces and interactors.

## 4. Methods

We obtained the list of human HTT interactors from the HIPPIE database [14]. For each interactor, we looked for its orthologous sequences in nine species, using the tool Proteinortho [42] with default parameters. The species we used were: *Pan troglodytes*, *Mus musculus*, *Rattus norvegicus*, *Sarcophilus harrisii*, *Gallus gallus*, *Anolis carolinensis*, *Xenopus tropicalis*, *Danio rerio*, and *Takifugu rubripes*. Their complete reference proteomes and the proteome set of 20,609 human proteins were downloaded from the UniProtKB database [43].

Multiple sequence alignments were obtained using MAFFT with default parameters [44]. A major technical problem that affects any evolutionary approach such as the one presented here is the existence of many protein sequences in the database that are likely fragments of the real sequence or have mistranslated regions. Given our focus on HTT, we manually curated the alignment of human HTT with orthologs. To do this we substituted sequences that seemed to be missing large parts at the start or end, or had large insertions or deletions, where these variations affected one sequence and were large, by alternative sequences from the NCBI RefSeq database [45].

Intrinsically disordered regions (IDRs) were computed using IUPred2 [46] with default parameters (setting long) and by taking regions of at least 30 amino acids. Coiled coil (CC) coordinates were downloaded from UniProt (as computed with the program COILS [47]). PolyX regions were computed using a threshold of 8 amino acids of the same type found in a window of 10 (following [48]). Amino acid-rich regions were computed with CAST2 using the default matrix BLOSUM62 and the default threshold of 40 [39]. 

The algorithm to consolidate LCRs in an alignment (IDRs, CCs, CBRs, and polyX) and describe their presence or absence worked as follows. For each aligned group of orthologs, the positions of each LCR are translated to positions in the multiple sequence alignment (taking gaps into account). Given an alignment and one type of feature (e.g., A-rich CBRs), the union of positions covered by the feature in any sequence is produced. This defines a series of intervals. Each interval defines a consolidated feature, with corresponding coordinates in the alignment, and a profile (binary vector) indicating if the interval contains a feature for each of the orthologs in the alignment.

The similarity scores between features (IDRs, CCs, amino acid-rich CBRs, and polyX) were computed in the following way. A binary vector of presence or absence of each feature in each interactor and ortholog was produced. Each pair of features is scored by the Jaccard similarity score between their vectors (number of vector entries with a 1 in both vectors divided by the number of vector entries with a 1 in any of the two vectors).

To cluster the HTT fragments and WW-domain interactors according to their IDR conservation, we converted their aligned sequences into a binary vector indicating whether each position in the alignment corresponds to (i) a residue predicted to be in an IDR, or (ii) something else: a residue not predicted to be in an IDR or a gap. Then, all possible pairs of sequences in the alignment are evaluated by the Jaccard similarity score, and these values constitute a vector for the corresponding HTT fragment or WW-domain interactor. These vectors were then hierarchically clustered using the R functions dist (Euclidean distance) and hclust (complete linkage).

## Figures and Tables

**Table 1:** Gene Ontology terms enriched in 402 human HTT-interactors.

Term	Count	*p*-Value
protein binding	315	2.28 × 10^−33^
myelin sheath	41	6.12 × 10^−30^
cytosol	153	1.36 × 10^−20^
mitochondrion	86	4.07 × 10^−18^
cytoplasm	179	5.81 × 10^−11^
membrane	99	1.32E × 10^−10^
extracellular exosome	116	1.82 × 10^−10^
nucleoplasm	114	5.51 × 10^−10^
ubiquitin protein ligase binding	31	1.93 × 10^−9^
identical protein binding	50	1.29 × 10^−8^
protein *C*-terminus binding	23	1.02 × 10^−7^
protein complex	33	1.27 × 10^−7^
protein complex binding	24	1.94 × 10^−7^
autophagosome	14	3.48 × 10^−7^
protein stabilization	20	4.17 × 10^−7^
cell–cell adherens junction	27	3.10 × 10^−6^
transcription factor binding	26	4.79 × 10^−6^
cadherin binding involved in cell–cell adhesion	26	7.30 × 10^−6^
mitochondrial inner membrane	31	1.02 × 10^−5^
macroautophagy	14	2.71 × 10^−5^

**Table 2:** The number of low complexity regions (LCRs) detected in 402 human HTT-interactors and orthologs in 10 species.

CCs	422	IDRs	658
total CBRs	1035	total polyX	236
A-rich	76	polyA	30
C-rich	3	polyC	0
D-rich	36	polyD	10
E-rich	201	polyE	46
F-rich	2	polyF	0
G-rich	71	polyG	28
H-rich	12	polyH	3
I-rich	4	polyI	0
K-rich	94	polyK	9
L-rich	4	polyL	11
M-rich	4	polyM	0
N-rich	7	polyN	0
P-rich	181	polyP	34
Q-rich	98	polyQ	27
R-rich	27	polyR	0
S-rich	182	polyS	33
T-rich	28	polyT	5
V-rich	3	polyV	0
W-rich	1	polyW	0
Y-rich	1	polyY	0

**Table 3:** LCRs detected in HTT and HAP40. Columns indicate feature type, coordinates in alignment, and conservation in 10 orthologs (as a binary pattern, see Methods for details).

**HTT**	**Coordinates**	**Conservation**
IDR	1–98	1111100000
polyQ	18–39	1101000000
CBR Q	18–69	1111100000
CBR A	22–83	0000001000
polyP	26–58	1111100000
CBR P	26–90	1111100000
polyP	65–82	1111100000
CBR S	398–705	1111111111
IDR	437–714	1111111111
CBR S	1033–1312	1111100011
IDR	1245–1305	1111111111
CBR P	2171–2193	0000000010
CBR E	2732–2746	1111100100
IDR	2732–2763	1111111100
polyE	2733–2743	1111000000
**HAP40**	**Coordinates**	**Conservation**
CBR G	15–88	0000--1000
CBR A	111–347	1111--1000
IDR	285–342	1111--0000
CBR P	291–342	1111--0000
polyP	305–314	1100--0000

## Data Availability

All data relevant to this study are provided as supplementary material.

## Supplementary Materials

The following are available online at https://www.mdpi.com/1422-0067/22/4/1727/s1, File S1: Gene Ontology terms enriched in 402 HTT-interactors. File S2: Numbered groups of orthologs used. Columns indicate: number of group (sorted by HIPPIE score of interaction of the human protein with HTT), ortholog identifiers in UniProt or “-“ if missing (for species Hsa *Homo sapiens*, Ptr *Pan troglodytes*, Mmu *Mus musculus*, Rno *Rattus norvegicus*, Sha *Sarcophilus harrisii*, Gga *Gallus gallus*, Aca *Anolis carolinensis*, Xtr *Xenopus tropicalis*, Dre *Danio rerio*, and Tru *Takifugu rubripes*), current UniProt identifier of the human protein (it might differ from the one in the original orthologous group), WW (y if the protein contains WW domains), SH3 (y if the protein contains SH3 domains), score (HIPPIE score of the interaction with HTT), and cluster (number of the cluster for WW proteins in Figure 6). File S3: Compressed file with sequences of orthologs used. File S4: Compressed file with multiple sequence alignments used. File S5: CCs detected in HTT-interactors. File S6: IDRs detected in HTT-interactors. File S7: CBRs detected in HTT-interactors. File S8: PolyX detected in HTT-interactors. File S9: Consolidated LCR features detected in HTT-interactors. File S10: Enrichment of LCRs in 402 human HTT-interactors. File S11: Table with the co-occurrence of features in HTT-interactors. File S12: Profiles of IDR conservation between pairs of orthologs for (i) WW domain-containing HTT-interactors or (ii) HTT fragments. File S13: Contains supplementary Figures S1 (number of LCRs by patters), S2 (most frequent conservation patterns of LCRs by amino acid type) and S3 (LCR co-occurrence matrix).
