# Supplementary material for: The Role of Low Complexity Regions in Protein Interaction Modes: An Illustration in Huntingtin
Source: Int J Mol Sci. 2021 Feb 9;22(4):1727. doi: 10.3390/ijms22041727 (PMC7915032; doi:10.3390/ijms22041727)
Supplement: Supplementary file 1 [file ijms-22-01727-s001.zip › Suppl_FileS13_SupplFigures.docx]

**The role of low complexity regions in protein interaction modes: an illustration in Huntingtin**

**Supplementary Figures**

| **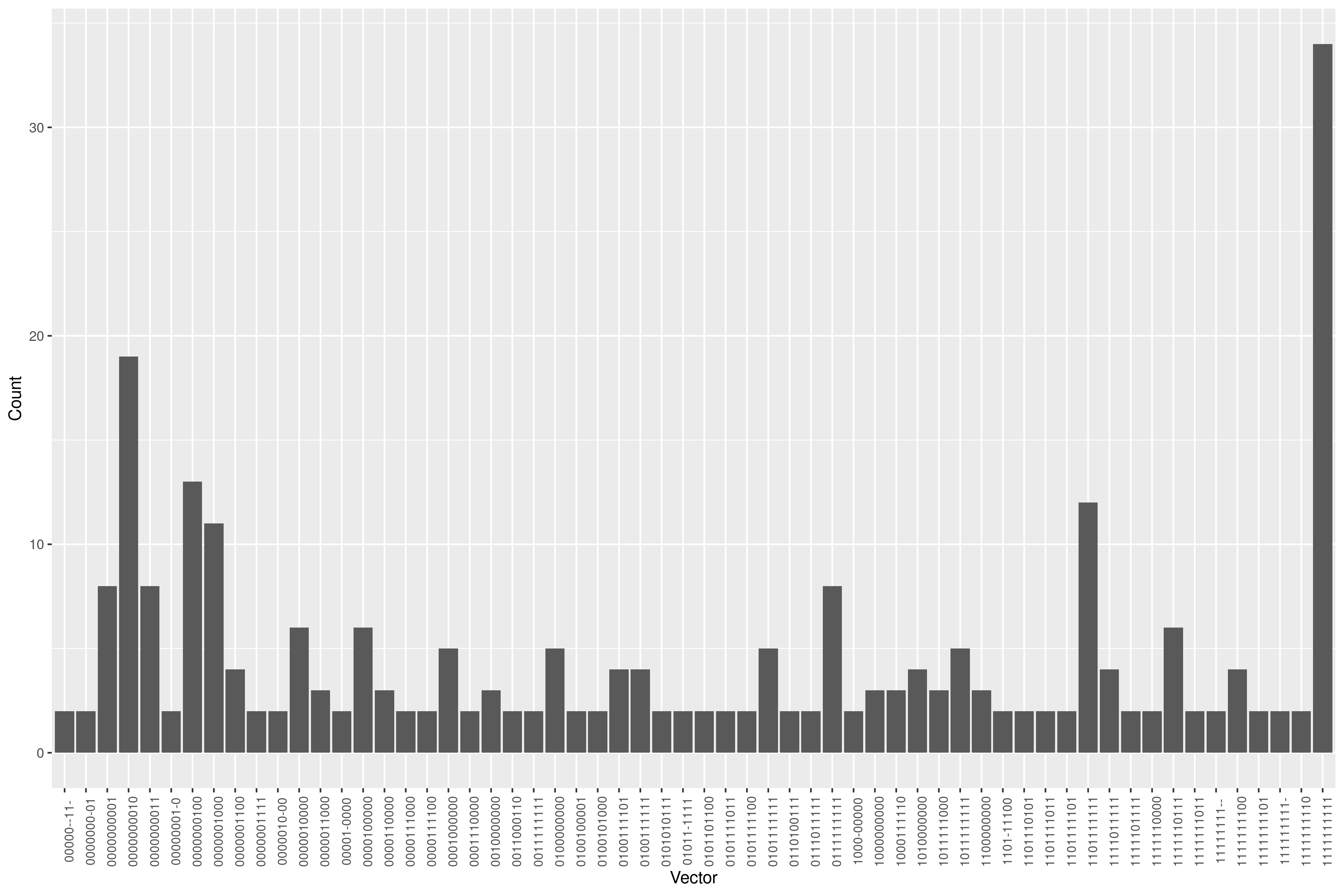** |
| --- |
| **(A)** |
| **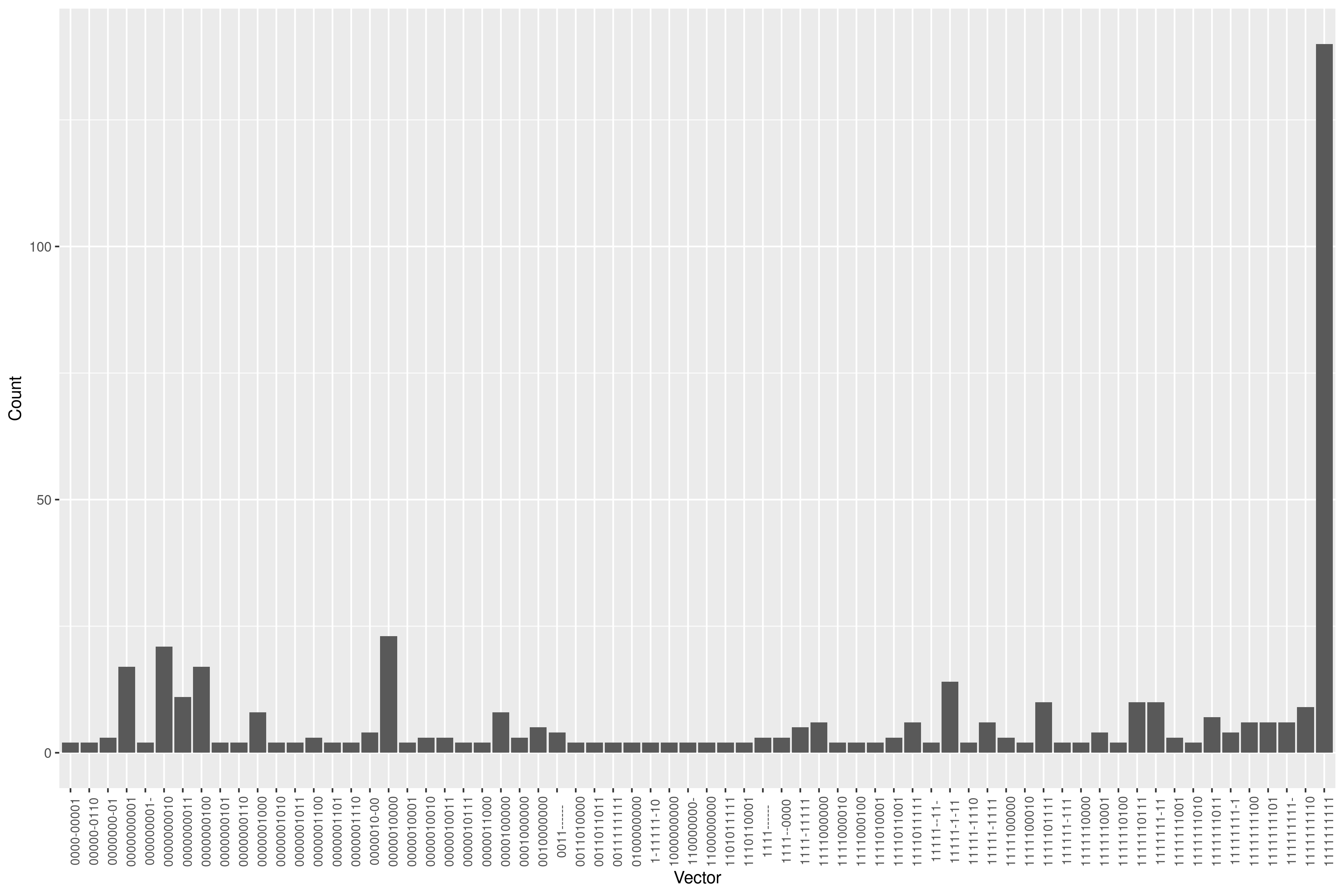** |
| **(B)** |
| **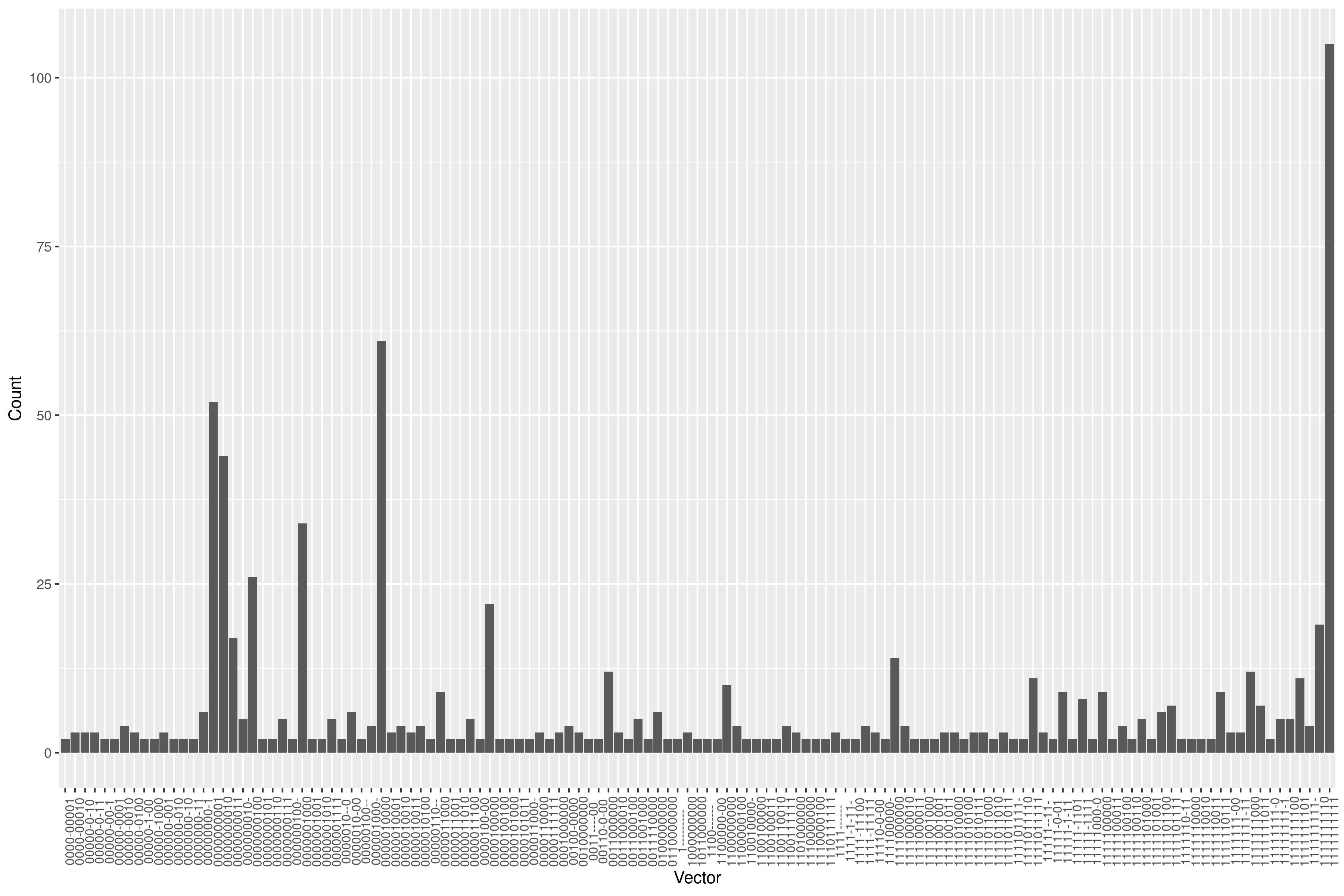** |
| **(C)** |
| **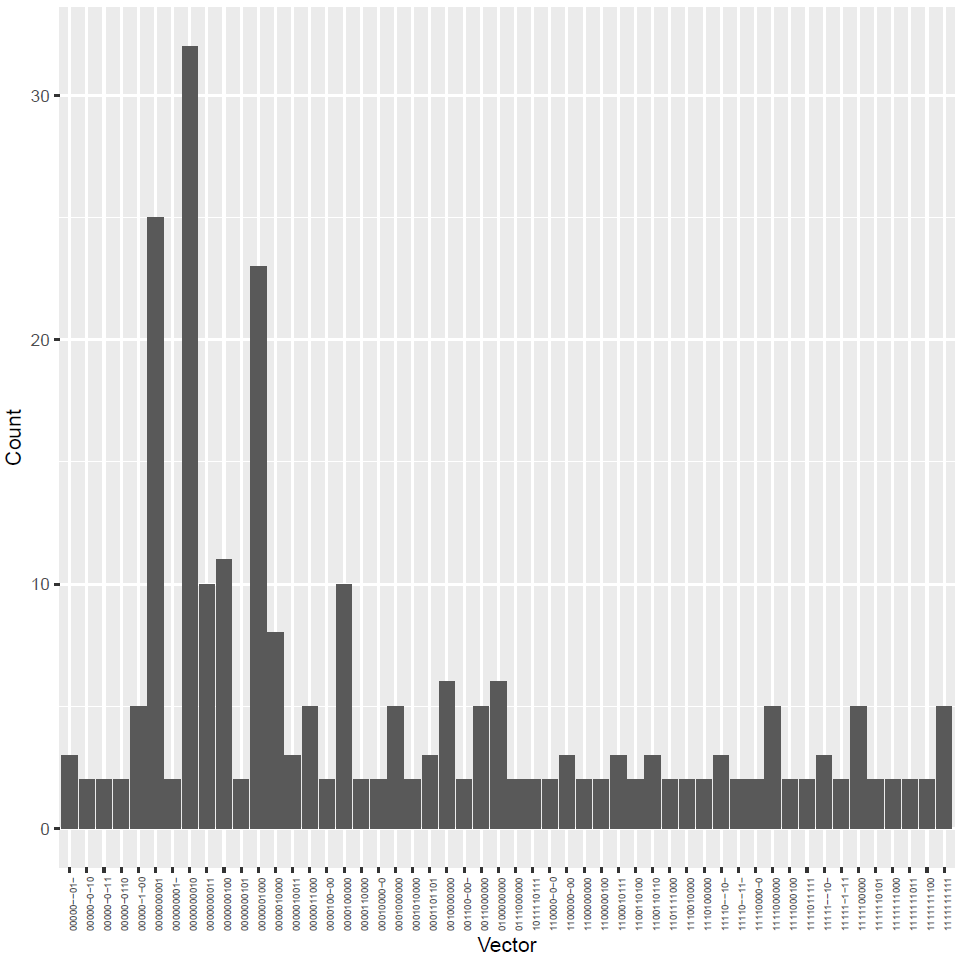** |
| **(D)** |

**Figure 1.** Number of LCRs by pattern. Note that a given LCR may occur more than once in a protein sequence. **(A)** CCs, **(B)** IDRs, **(C)** CBRs, **(D)** polyX. Patterns are sorted alphabetically. Only patterns occurring more than once are included. The binary patterns represent the conservation of the corresponding feature in species: *Homo sapiens, Pan troglodytes, Mus musculus, Rattus norvegicus, Sarcophilus harrisii, Gallus gallus, Anolis carolinensis, Xenopus tropicalis, Danio rerio, Takifugu rubripes*. Values are “1” for present in ortholog, “0” for absent in ortholog, and “-“ if ortholog was missing.

| **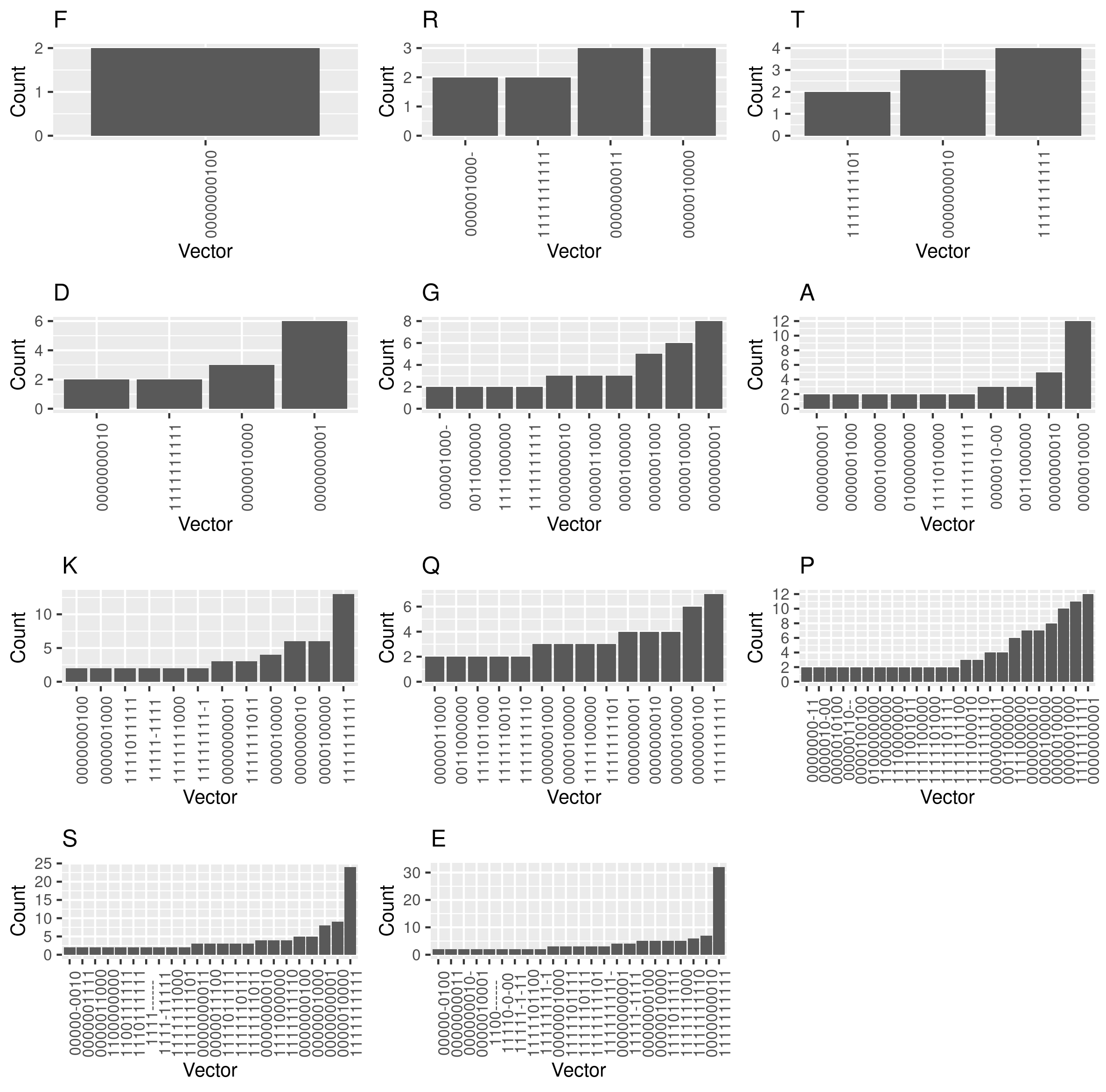** |
| --- |
| (**A**) |
| 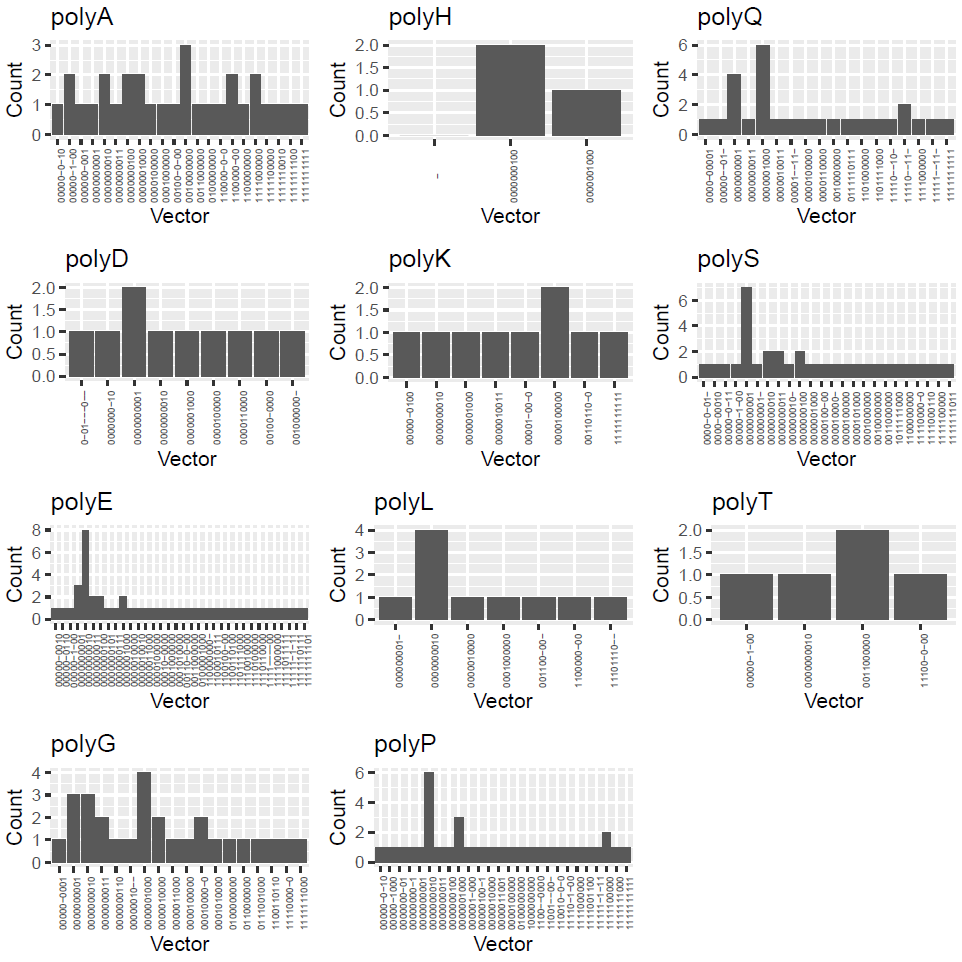 |
| (**B**) |

**Figure S2.** Most frequent conservation patterns of LCRs by amino acid type. **(A)** CBRs (occurring at least twice). **(B)** PolyX. Binary patterns defined as for Figure S1.


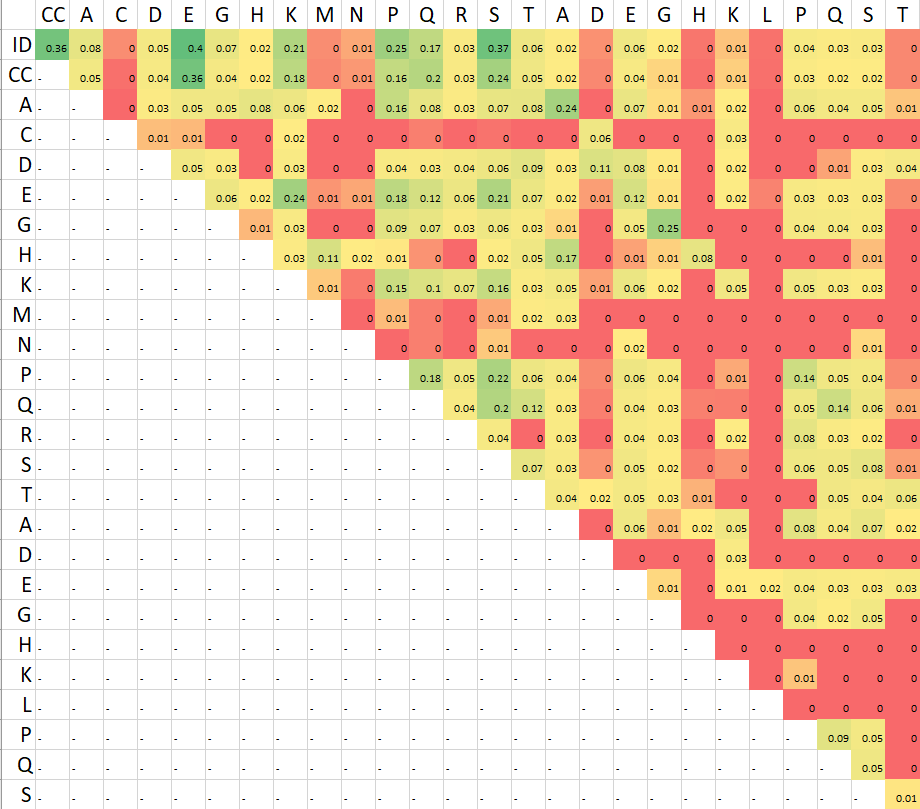


polyX

CBRs

polyX

CBRs

**Figure S3.** LCR co-occurrence matrix. Co-occurrence between LCR types was calculated across all sequences of HTT interactors and orthologs (see Methods for details). The color scale represents values from the highest in green (IDRs to E-rich, 0.40), through middle values in yellow, down to the lowest in red (zero). LCRs were included in this representation if they have at least one score of co-occurrence with another LCR above 0.02. See Methods for details about the calculation of the scores. Raw values are available as Supplementary File S11. The black boxes highlight co-occurrences of CBRs and polyX, respectively.
